# Trajectories of class–switching‐related egg and cow's milk allergen‐specific immunoglobulin isotype formation and its modification by eczema with low‐ and high‐affinity immunoglobulin E during early infancy

**DOI:** 10.1002/iid3.245

**Published:** 2019-03-11

**Authors:** Makoto Irahara, Wakako Shinahara, Mayumi Sugimoto, Yukiko Ogawa, Keiji Shitsukawa, Kenji Kubota, Limin Yang, Yukihiro Ohya, Hirohisa Saito, Shoji Kagami, Kokichi Arisawa, Hiroshi Kido

**Affiliations:** ^1^ Division of Enzyme Chemistry Institute for Enzyme Research, Tokushima University Tokushima Japan; ^2^ Department of Pediatrics Graduate School of Medicine, Tokushima University Tokushima Japan; ^3^ Department of Pediatrics Tokushima Prefecture Naruto Hospital, Naruto Tokushima Japan; ^4^ Department of Obstetrics and Gynecology Tokushima Prefecture Naruto Hospital, Naruto Tokushima Japan; ^5^ Division of Allergy, Department of Medical Subspecialties National Center for Child Health and Development, Okura Setagaya‐ku Tokyo Japan; ^6^ Department of Preventive Medicine Graduate School of Medicine, Tokushima University Tokushima Japan

**Keywords:** allergen microarray, eczema, food allergy, high‐affinity immunoglobulin E, immunoglobulin isotype formation, low‐affinity immunoglobulin E

## Abstract

**Introduction:**

Allergen‐specific immunoglobulin isotype formation associated with immunoglobulin class‐switching during the lactation period is the immunological background for food allergy in infants. We analyzed the serial changes in the production of feeding type‐related egg‐ and milk‐specific immunoglobulin isotypes from birth to 6 months of age with or without eczema in 84 infants.

**Methods:**

Allergen‐specific immunoglobulin G1 (IgG1), IgG2, IgG3, IgG4, IgA, and IgE levels of hen's egg and bovine milk were measured in cord blood and blood samples from infants at 2, 4, and 6 months of age by the densely carboxylated protein microarray.

**Results:**

Formula and mixed feeding were associated with a rapid increase in cow's milk allergen‐specific immunoglobulins and feeding type‐related significant differences in casein‐specific immunoglobulin levels were detected. Breast and mixed feeding were associated with slow but significant increase in ovalbumin‐specific IgG1 and IgE levels, but not other immunoglobulins. We found two different immunoglobulin isotype formation at 6 months of age with low‐ or high‐affinity IgE against ovalbumin. One isotype formation pattern had relatively high ovalbumin‐specific IgG1 levels, detectable IgG2, and low‐affinity IgE, while the other had low ovalbumin‐specific IgG1 levels, undetectable IgG2, and high levels of high‐affinity IgE. The incidence of eczema was significantly higher in the latter pattern (84.6%), compared with the remaining infants (42.2%).

**Conclusions:**

Feeding practice‐related allergen sensitization and immunoglobulin isotype formation were identified during the lactation period. The development of eczema during the lactation period could potentially modify the immunoglobulin isotype formation with high levels of high‐affinity IgE.

## 1 INTRODUCTION

Most people have lifelong clinical and immunological tolerance to various food items, but a minority (approximately 3%‐6%) suffers from adverse reactions^1^. Although various food items can potentially induce immunoglobulin E (IgE)‐mediated food allergy, allergies to hen's egg and cow's milk allergens are the earliest to appear and most common food allergies in infants.^1^, ^2^ These food allergies often appear in the early stage of the allergic march,^3^ along with atopic dermatitis, and are associated with increased risks of anaphylaxis and asthma.^4^

Early introduction of food seems more beneficial in reducing the incidence of food allergy than late introduction or avoidance of food allergens,^5^, ^6^, ^7^, ^8^ although one study reported that egg introduction at 4 months of age in infants with moderate‐to‐severe eczema provided insufficient protection and resulted in the appearance of various problems.^9^ However, we reported recently that introduction of heated egg in a stepwise manner together with aggressive treatment of eczema is a safe and effective approach in preventing hen's egg allergy at 1 year of age in high‐risk infants.^10^ In addition, animal studies have shown that the development of oral tolerance is driven by allergen exposure during a critical early window of development,^11^ although the time frame of this potential window is not clear in human subjects. Several birth cohort studies concluded that delayed introduction of specific food items (eg, egg white, cow's milk, fish, and oats) beyond the 6 to 9 months of age is associated with increased risk of allergic disease.^12^, ^13^, ^14^ These results suggest that the association between the development of immunological response and food allergen sensitization during the lactation period in early infancy is an important background of food allergy and oral tolerance. In addition, accumulating evidence indicates the presence of a strong association between food sensitization and atopic dermatitis and/or skin barrier impairment in breastfed infants, suggesting that allergic sensitization to foods can be mediated by cutaneous antigen‐presenting cells.^15^, ^16^

It has been reported that the presence of food proteins in human breast milk, such as ovalbumin (OVA),^17^, ^18^, ^19^, ^20^ bovine *β*‐lactoglobulin,^17^, ^20^ gliadin^21^ and peanut proteins,^22^ and breast milk‐mediated transfer of antigens to the neonate results in the induction of oral tolerance.^23^, ^24^ To understand the development of food allergy and its relationship with eczema during the lactation period, there is a need for a trajectory study on allergen‐specific immunoglobulin production associated with immunoglobulin class‐switching recombination (*μ* → *γ*3 → *γ*1 → *γ*2 → *γ*4, and *γ*1 → *ε*) from birth to 6 months of age. To our knowledge, no such detailed data are available at present, probably due to the methodological limitation of quantitating the instrument of allergen‐specific IgG1, IgG2, IgG3, IgG4, IgE, and IgA by using small amounts of blood from infants. To monitor immunoglobulin isotype production, we developed recently a highly sensitive densely carboxylated protein (DCP) microarray with high antigen immobilization capacity.^25^, ^26^, ^27^ The DCP microarray offers the advantages of measurement of various immunoglobulin isotypes under the same assay conditions by using a small volume of serum or plasma (100 μL for all analyses). In addition, we reported recently that DCP microarray can detect allergen‐specific low‐ and high‐affinity IgE isotypes and high‐affinity IgE, but not low‐affinity IgE, that can elicit mast cell degranulation.^28^

The aim of the present study was to monitor the serial changes in the formation of feeding type (formula, breast, and mixed feeding)‐related allergen‐specific immunoglobulin isotypes during early infancy. We measured allergen sensitization associated with immunoglobulin class‐switching from IgG1 to IgE and/or IgG2 in each infant against OVA, the major hen's egg allergen and casein, the major bovine milk allergen by the DCP microarray. We also searched for any modification in immunoglobulin isotype formation associated with eczema in producing immunoglobulin isotypes with allergen‐specific low‐ or high‐affinity IgE.

## 2 MATERIALS AND METHODS

### 2.1 Study design and study samples

**Figure 1:**
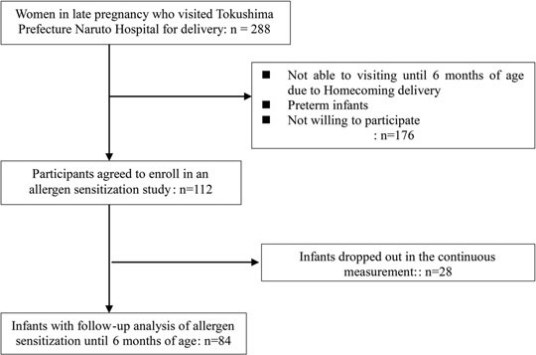
Study enrollment profile

Among the healthy pregnant women who visited Tokushima Prefecture Naruto Hospital for delivery between November 2013 and October 2014 and agreed to join this study, the present study enrolled 112 of their infants. The applied exclusion criteria were birth before 37 weeks of gestation and complications associated with any chronic or severe disease. Primary‐outcome data were obtained in 84 (75%) of the 112 eligible participants whereas serial blood sampling could not be achieved in the dropout infants (n = 28) for a variety of reasons such as change of address after childbirth and will of no further blood sampling (Figure 1 and Table 1).

**Table 1:** Characteristic of participants

Total number of study subjects	84
Gestational age, d	274 ± 7
Birth weight, g	3067 ± 364
Sex	
Males	42 (50%)
Females	42 (50%)
Feeding type until 6 mo of age	
Breast milk feeding	31 (37%)
Formula milk feeding	6 (7%)
Mixed feeding	47 (56%)
Eczema until 6 mo of age^a^	
Eczema	41 (49%)
No skin eczema	43 (51%)

Data are mean ± SD or number (percentage) of patients.

^a^ A pediatric allergologist examined the infants to assess their skin condition according to the UK working criteria.^29^

### 2.2 Clinical data collection

A pediatric allergologist examined the infants at 2, 4, and 6 months of age to assess the skin condition and performed blood sampling. In contrast, cord blood (CB) samples were collected by the obstetricians at birth by needle puncture of the umbilical vein after careful cleaning of the umbilical cord to avoid maternal blood contamination. CB and blood samples were collected from the infants at 2, 4, and 6 months of age, centrifuged at 1500*g* for 10 minutes and frozen at −30°C until analysis. We confirmed histories of parental allergic disease by check list in prenatal periods and the state of birth at 2 months of age. Eczema was diagnosed according to the criteria of the UK working group^29^ and information on feeding formula milk, breast milk, and mixed food was obtained from the mothers in each visit.

### 2.3 Measurement of allergen‐specific immunoglobulin isotypes

Hen's egg and cow's milk allergen‐specific IgE, IgG1, IgG2 IgG3, IgG4, and IgA levels in blood were measured by the DCP microarray as described in detail previously.^25^ The individual arrays were incubated for 2 hours with 20 μL of 1:5 (IgE, IgA) or 1:50 (IgG1, IgG2, IgG3, IgG4) diluted plasma, washed three times, and then reacted with a HiLyte Fluor 555 (Dojindo Molecular Technologies, Inc, Kumamoto, Japan)‐labeled secondary monoclonal antibody against each human immunoglobulin for 2 hours. The resulting images were acquired by scanning the microarrays with a 3D‐Gene Scanner 3000 (Toray, Tokyo, Japan), and converted to numerical fluorescence intensity. Using standard concentrations of purified IgE (Abbiotec, San Diego, CA) and IgGs and IgA (Bio‐Rad, San Francisco, CA), the amounts of allergen‐specific antibodies bound to the microarrays were calculated and expressed as binding units (BU). The BUs of IgE, IgA, and IgG subclasses were reported as BUe, BUa, and BUg1‐4, respectively. The detection limits for OVA‐ and casein‐specific IgE, IgA, IgG1, IgG2, IgG3, and IgG4 are 10.0 BUe/mL, 2.5 BUa/mL, 100.0 BUg1/mL, 50.0 BUg2/mL, 50.0 BUg3/mL, and 50.0 BUg4/mL, respectively, under the present assay conditions. Data of allergen‐specific IgE analyzed by the DCP biotechnique correlate strongly with allergen‐specific IgE values determined for various allergens with the UniCAP system (*r* > 0.9‐0.85).^25^ Allergen‐specific IgE values of the DCP system (BUe/mL) are able to convert to those of the UniCAP system (kU_A_/L) by each allergen‐specific equation. For instance, correlation coefficient of casein‐specific IgE between UniCAP (kU_A_/L) and DCP microarray (BUe/mL) and its equation are shown in Figure S1. Based on the small volume of the blood sample and wide assay dynamic range, the DCP assay method has been used in several cohort studies.^10^, ^26^, ^27^, ^28^, ^30^

### 2.4 Measurement of affinity of OVA‐specific immunoglobulin E

The OVA affinity of IgE was analyzed using an OVA‐immobilized DCP microarray, which is based on competition binding inhibition of serially diluted soluble OVA, and expressed as IC_50_ (nM), which represented the concentration of allergen required for 50% inhibition, as described previously.^28^ For the analysis, IgE levels must be higher than 200 BUe/mL to evaluate any subsequent suppression in their values. Briefly, OVA in phosphate‐buffered saline was added to the plasma samples at final concentrations of 0 to 0.1 × 10^−6^ mol/L and then incubated at 25°C for 30 minutes. After incubation, each reaction mixture was applied to an OVA‐immobilized DCP microarray and the competitive binding assay was conducted at 37°C for 60 minutes. After washing three times, the bound antibody was detected and then IC_50_ was calculated.

### 2.5 Statistical analysis

Differences in allergen‐specific immunoglobulins among formula milk, breast milk, and mixed‐feeding infants at each time point were analyzed by the Kruskal‐Wallis test. Differences in continuous variables of allergen‐specific immunoglobulins at four different time points (ie, at birth, 2, 4, and 6 months of age), and differences between two time points were analyzed by the Friedman test and the Wilcoxon signed‐rank test with Bonferroni's correction, respectively. We evaluated IC_50_ values of OVA‐specific IgE and cumulative incidence of eczema between the red circled infants in group A and black circled infants in group B in Figure 6 by the Wilcoxon rank‐sum test and the Fisher's exact test, respectively. *P* < 0.05 was considered significant in all analyses except for the Wilcoxon signed‐rank test with Bonferroni's correction. *P* < 0.01 (0.05/predetermined number of comparisons) was judged to be significant for the Wilcoxon signed‐rank test with Bonferroni's correction. All statistical analyses were performed using SPSS for Windows (version 22) (IBM, New York, NY).

### 2.6 Ethics statement

This study was conducted according to the principles in the Declaration of Helsinki. This study was approved by the Ethics Committee of Tokushima Prefecture Naruto Hospital (Permit #1314) on 14th November 2013. Written informed consent was obtained from each mother at the time of enrollment. This study was carried out in accordance with relevant guidelines and regulations.

## 3 RESULTS

### 3.1 Participants

The primary‐outcome data were obtained from 84 healthy infants in the studies on immunoglobulin isotype production in early infancy (Figure 1). Blood samples were collected every 2 months from each infant. Table 1 summarizes the baseline characteristics of the participants. Throughout the 6‐month study, 31 participants were on breast milk, six on formula milk, and 47 on mixed feeding (breast milk and formula milk). Among the participants, 41 infants developed eczema during the study period (16 [51.6%] on breast milk, 2 [33.3%] on formula milk, and 23 [48.9%] on mixed feeding). Eczema was diagnosed by a pediatric allergologist. Based on interviews with the parents, none of the infants was offered hen's egg or experienced adverse reactions to any food during the study period.

### 3.2 Serial changes in feeding type‐related production of allergen‐specific immunoglobulin isotypes

**Figure 2:**
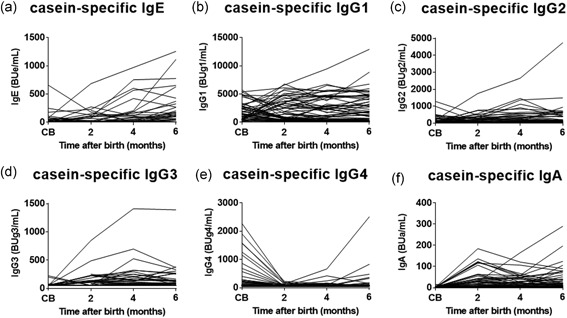
Serial changes in casein‐specific immunoglobulin isotype formation during the first 6 months of life. Casein‐specific immunoglobulin E (IgE) (A), IgG1 (B), IgG2 (C), IgG3 (D), IgG4 (E), and IgA (F) levels in cord blood (CB) and blood samples obtained at 2, 4, and 6 months of age analyzed by the densely carboxylated protein microarray

**Figure 3:**
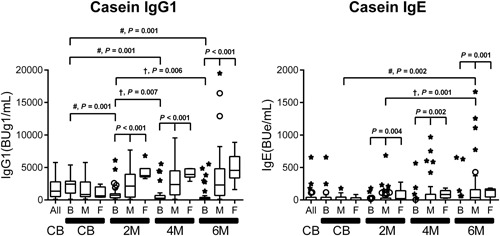
Serial changes in feeding type‐related casein‐specific immunoglobulin G1 (IgG1) and IgE production in infants on breast milk (B), formula milk (F), and mixed (M) feeding from birth to 6 months of age. Data are the median (interquartile range) values of casein‐specific IgG1 and IgE in cord blood (CB), and in plasma at 2, 4, and 6 months (2 M, 4 M, and 6 M). In these box‐and‐whisker plots, lines within the boxes represent the median values; the upper and lower lines of the boxes represent the 25th and 75th percentiles, respectively; and the upper and lower bars outside the boxes represent the 90th and 10th percentiles, respectively. For statistical analysis, the CB samples were retrospectively divided into three corresponding groups. Differences among the three groups were analyzed by the Kruskal‐Wallis test. The differences (# and †) between two time points are described in Table S1: the Wilcoxon signed‐rank test with Bonferroni's correction. No significant difference was detected unless otherwise marked

Production of allergen‐specific immunoglobulins in blood is highly dependent on the dose of allergens in the ingested milk. Since casein accounts for about 80% of the total cow's milk proteins,^31^ dietary intake of casein in the formula and mixed feeding stimulates the induction of rapid production of casein‐specific immunoglobulin isotypes in infants in the early stage of lactation. In addition, human breast milk also contains low levels of cow's milk allergen, *β*‐lactoglobulin.^32^ Table S1 shows the results of statistical analysis of feeding type‐related casein‐specific IgE, IgG1, IgG2, IgG3, IgG4, and IgA production in infants on breast, formula, and mixed feeding from birth to 6 months of age. Feeding type‐related significant differences in the levels of each casein‐specific immunoglobulin were detected among the three groups at 2, 4, and 6 months of age, with the exception of IgG4 at 2 months (Table S1); the highest levels were in infants on formula feeding, followed by infants on mixed feeding while the lowest levels were in infants on breast milk. Figure 2 shows the serial changes in the production of casein‐specific immunoglobulin isotypes during the first 6 months of life. Since maternal IgG, but not IgA and IgE, are transferred to the fetus across the placenta through a receptor‐mediated mechanism,^27^, ^33^, ^34^ high levels of casein‐specific IgG1 and IgG4 among immunoglobulins were found in the CB but their levels rapidly and significantly fell particularly in infants on breast milk after 2 months of age (Table S1). Furthermore, a rapid rise in casein‐specific IgG1 level was detected after birth and plateaued at 2 months of age (Figure 2), without any further significant increase in the formula and mixed feeding groups (Table S1 and Figure 3). A similar increase was also noted in casein‐specific IgE in infants on formula and mixed feeding, but the increase was only statistically significant in infants on mixed feeding, not in the other infants, at 6 months of age (Figure 3 and Table S1). We also identified time‐dependent increases in other casein‐specific immunoglobulins, such as IgG2, IgG3, IgG4, and IgA, in infants on formula and mixed feeding, but they were not statistically significant until 6 months of age. The levels of casein‐specific IgG4 and IgA started to increase at 6 months of age in some infants (Figure 2). Similar results were found with other milk allergens, such as milk allergen‐ and *β*‐lactoglobulin‐specific immunoglobulins (data not shown).

**Figure 4:**
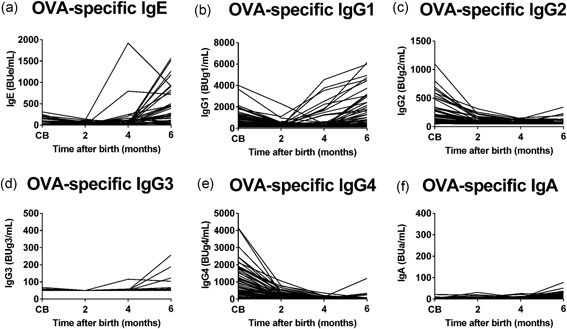
Serial changes in ovalbumin (OVA)‐specific immunoglobulin isotype formation from birth to 6 months of age. OVA‐specific immunoglobulin E (IgE) (A), IgG1 (B), IgG2 (C), IgG3 (D), IgG4 (E), and IgA (F) levels in cord blood (CB) and blood samples obtained at 2, 4, and 6 months of age were analyzed by the densely carboxylated protein microarray

**Figure 5:**
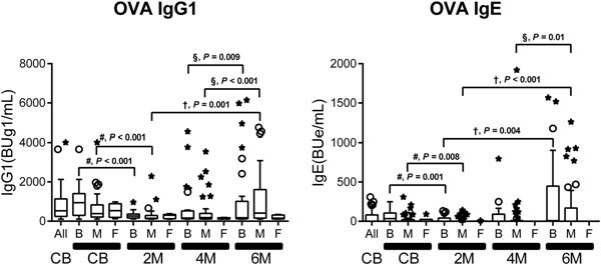
Serial changes in feeding type‐related ovalbumin (OVA)‐specific immunoglobulin G1 (IgG1) and IgE production in infants on breast milk (B), formula milk (F), and mixed (M) feeding from birth to 6 months of age. The median (interquartile range) values of OVA‐specific IgG1 and IgE in cord blood (CB) and in plasma at 2, 4, and 6 months (2 M, 4 M, and 6 M). The difference #, vs CB, †, vs 2 months of age, and § vs 4 months of age, by the Wilcoxon signed‐rank test with Bonferroni's correction as described in Table S2. No significant difference was detected unless otherwise marked

In contrast to the relatively high concentration of casein in formula milk, the average breast milk OVA concentration is less than 1 ng/mL,^19^ although the concentration depends on the amount of maternal egg ingestion. The breast and mixed feeding slowly induced OVA‐specific immunoglobulin isotype production in infants during the lactation period. The pattern of OVA‐specific immunoglobulin isotype production during the study period was different from that of casein‐specific immunoglobulin production (Figure 4). High levels of OVA‐specific IgG1, IgG2, and IgG4 were found in CB but precipitously decreased by 2 or 4 months of age. Interestingly, the feeding type had no significant impact on the levels of OVA‐specific immunoglobulins regardless of age after birth (Figure 5 and Table S2), with the exception of IgG1 and IgA at 2 months of age. In contrast, OVA‐specific IgG1 and IgE levels increased significantly with breast and mixed feeding, as determined in 6‐month‐old infants (Figure 5 and Table S2). However, there was no significant time‐dependent increase in other OVA‐specific immunoglobulins, such as IgG2, IgG3, IgG4, and IgA, even in infants on breast milk up to 6 months of age. Similar results were found with other egg allergens, such as egg white‐ and ovomucoid‐specific immunoglobulins (data not shown).

### 3.3 Serial changes in correlations between casein‐ and OVA‐specific IgG1 and IgE production

**Figure 6:**
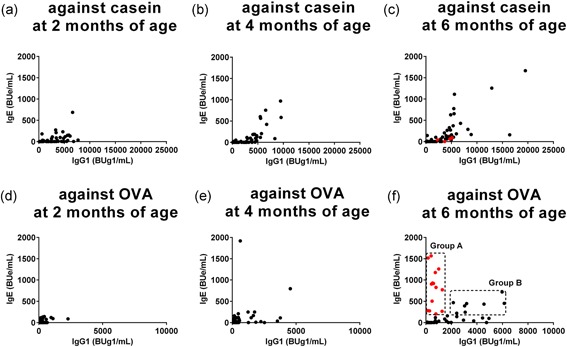
Serial changes in correlations between casein‐ and ovalbumin (OVA)‐specific immunoglobulin G1 (IgG1) and IgE levels in each infant group from 2 to 6 months of age. Changes in correlations between IgE and IgG1 levels in blood samples against casein (A‐C) and OVA (D‐F) were monitored in each infant group from 2 to 6 months of age. (F) Infants with IgE levels of greater than or equal to 200 BUe/mL were divided into red symbols infants in group A (n = 13), and black symbols infants in group B (n = 7), as described in the text

Serial changes in the production of allergen‐specific immunoglobulin isotypes are related to repeated antigen exposure and the results of immunoglobulin class‐switching recombination.^35^, ^36^ Figure 6 shows changes in casein‐ and OVA‐specific IgG1 and IgE levels in 2‐ to 6‐month‐old infants. Increased casein‐specific IgG1 levels were detected in the early phase of the lactation period after 2 months of age, and IgE appearance and its gradual increase were found after IgG1 levels exceeded at 2000 BUg1/mL in the majority of infants (Figure 6A‐C). The results suggest that allergen‐specific IgG1 accumulation is a prerequisite for IgE production.

OVA‐specific maternal IgG1 in CB almost disappeared at 2 months of age and then OVA‐specific IgG1 and IgE produced by infants increased slowly after 4 months of age (Figure 6D‐F). At 6 months of age, two groups of infants with different correlation between OVA‐specific IgG1 and IgE levels were noted. One group of infants had relatively low levels of OVA‐specific IgE production along with an increase in IgG1 levels exceeding at 2000 BUg1/mL in a manner similar to those found in casein‐specific IgE. The other group of infants had relatively low OVA‐specific IgG1 levels (<2000 BUg1/mL) and high IgE levels, although similar type was not detected in casein‐specific IgG1 and IgE correlation (Figure 6C and 6F). Next, we measured the affinity characteristics of these IgE against OVA in the two groups.

### 3.4 Detection of OVA‐specific low‐ and high‐affinity immunoglobulin E

**Figure 7:**
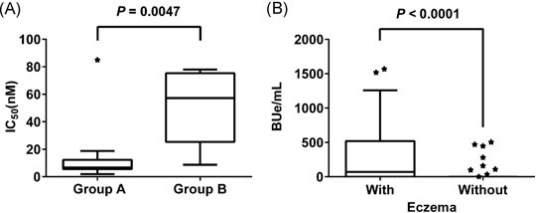
Detection of ovalbumin (OVA)‐specific low‐ and high‐affinity IgE in infants at 6 months of age (A) and OVA‐specific IgE levels in infants with or without eczema (B). A, OVA affinity of immunoglobulin E (IgE) in infants in groups A and B was analyzed using an OVA‐immobilized densely carboxylated protein microarray by competition binding inhibition and expressed as median values of IC_50_ (nM). See Table S3 for more details. B, Median values of OVA‐specific IgE levels in infants at 6 months of age with (n = 41) and without history of eczema (n = 43). Statistical analysis by Fisher's extract test

For measurement of allergen‐specific affinity of IgE by competitive binding inhibition, we used samples with IgE levels higher than 200 BUe/mL to monitor inhibition of binding and at least five different concentrations of allergen were used for competitive binding inhibition. Infants with IgE levels greater than or equal to 200 BUe/mL (Figure 6F) were divided into two groups. The first group (group A) included infants with OVA‐specific IgG1 levels of less than 2,000 BUg1/mL and IgE levels of greater than equal to 200 BUe/mL (Figure 6F, red circles enclosed within the rectangle, n = 13). The other group (group B) included infants with OVA‐specific IgG1 levels of greater than equal to 2000 BUg1/mL and IgE levels of greater than equal to 200 BUe/mL (Figure 6F, black circles enclosed within the rectangle, n = 7). The IC_50_ values (Figure 7A) and OVA‐specific IgE and IgG1 levels of these infants are shown in Table S3. The red‐circles infants in group A showed high‐affinity IgE with median (interquartile range) IC_50_ value of 6.68 (6.47‐9.24) nM, while those of the black circles in group B had low‐affinity IgE with IC_50_ value of 57.21 (35.34‐68.27) nM. The IC_50_ values of the two groups were significantly different (*P* = 0.0047).

The cumulative incidence of eczema history was significantly higher in the red‐circles infants in group A (84.6%, 11 out of 13 infants) compared with that of the remaining infants (42.2%, 30 out of 71 infants, *P* = 0.001). Figure 7B shows OVA‐specific IgE levels in infants at 6 months of age with (n = 41) or without (n = 43) eczema history. OVA‐specific IgE levels were significantly higher in infants with eczema history than in those without (*P* < 0.0001).

### 3.5 Different patterns of immunoglobulin isotype formation with low‐ and high‐affinity IgE in 6‐month‐old infants

**Figure 8:**
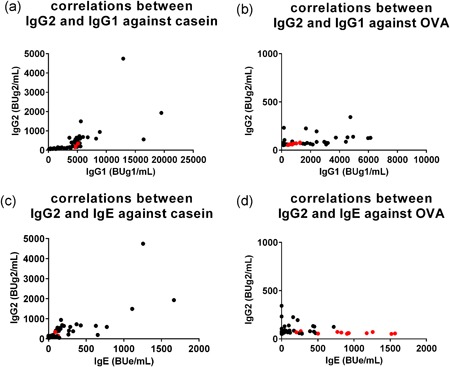
Correlations among immunoglobulin G1 (IgG1) and IgG2 and IgG2 and IgE against casein and ovalbumin (OVA) in infants at 6 months of age. Correlations between casein‐specific IgG1 and IgG2 levels (A), OVA‐specific IgG1 and IgG2 levels (B), casein‐specific IgE and IgG2 levels (C), and OVA‐specific IgE and IgG2 levels (D). See legend of Figure 6F for the explanation of the red and black circles

Since sequential immunoglobulin class‐switching of *γ*1 → *ε* and *γ*1 → *γ*2 has been reported,^35^, ^36^ we analyzed the correlations between OVA‐ and casein‐specific IgG1 and IgG2 levels and IgG2 and IgE levels at 6 months of age. Casein‐specific IgG2 production was detected along with increases in IgG1 levels of greater than or equal to 2,000 BUg1/mL, in a manner similar to casein‐specific IgE production in the majority of infants (Figure 8A). OVA‐specific IgG2 production in the majority of the black circles infants in group B, who had low‐affinity IgE, was also detected along with an increase in high IgG1 levels of greater than or equal to 2000 BUg1/mL (Figure 8B), although the production was less clear compared with casein‐specific IgG2 production. In contrast, undetectable production of OVA‐specific IgG2 was found in the red‐circles infants in group A, who showed high‐affinity IgE (Figure 8B). The correlations between OVA‐specific IgG2 and IgE (Figure 8D) showed undetectable IgG2 production in the red‐circles infants with high‐affinity IgE. These results suggest that the development of eczema during the lactation period could suppressively modify the immunoglobulin isotype formation associated with immunoglobulin class‐switching from IgG1 to IgG2 with the production of high‐affinity IgE.

## 4 DISCUSSION

In the present study, we elucidated the serial changes in feeding‐related egg and cow's milk allergen‐specific immunoglobulin isotype formation associated with immunoglobulin class‐switching process from birth to 6 months of age using the DCP microarray. The results showed that formula and mixed‐feeding infants (who ingested a high dose of cow's milk allergens^31^) showed an early immunoglobulin isotype formation; ie, induction of high levels of casein‐specific IgG1 from 2 months of age with no further significant increase up to 6 months of age. During this early sensitization and induction of IgG1, the levels of greater than or equal to 2000 BUg1/mL were associated with early and almost simultaneous production of casein‐specific IgE and IgG2, suggesting that the immunoglobulin class‐switching process progresses from *γ*1 to *ε* and *γ*1 to *γ*2, respectively, after 2 months of age (Figures 2 and 3 and Figures 6 and 8).

Breast and mixed‐feeding infants who were exposed to small amounts of egg white allergens,^19^ showed slow immunoglobulin isotype formation; ie, mild OVA‐specific IgG1 production after 2 months of age (Figures 4, 5), but this was coupled with significant gains at 6 months of age (Figure 5 and Table S2). OVA‐specific IgE production was detected after 4 months of age (Figure 4) and the significant gain was observed at 6 months of age compared with those at 2 or 4 months of age (Figures 5 and 6 and Table S2). In breast and mixed‐feeding infants, our results showed two patterns of OVA‐specific immunoglobulin isotype formation with high‐affinity IgE (red circles in group A) and low‐affinity IgE (black circles in group B) at 6 months of age (Figure 6F). In the black circles infants in group B, OVA‐specific IgG1 level of greater than or equal to 2000 BUg1/mL was accompanied by IgE and IgG2 production in a manner similar to casein‐specific IgE and IgG2 (Figures 6 and 8), although the induction of IgE and IgG2 was less evident than the casein‐specific induction of IgE and IgG2. The relatively low levels of OVA‐specific IgE in the black circles infants in group B showed low OVA‐affinity characteristics (Figure 7A). In contrast, the red circles infants in group A showed OVA‐specific immunoglobulin isotype formation with relatively low levels of IgG1 of less than 2000 BUg1/mL and high levels of IgE with undetectable IgG2 production (Figure 6F and Figure 8B and 8D). The OVA‐specific IgE in the red circles infants showed high OVA‐affinity characteristics (Figure 7A). Similar immunoglobulin isotype formation with relatively higher levels of IgE coupled with lower levels of IgG was recently reported in animal experiments.^37^ They reported that epicutaneously sensitized mice had higher levels of OVA‐specific IgE and lower levels of OVA‐specific IgG1, IgG2a, and secretory IgA than those found in intraperitoneally sensitized mice.

The cumulative incidence of eczema was significantly high in the red circles infants (84.6%), compared with that of 42.2% in the remaining infants (*P* = 0.001). This finding suggests that eczema or inflammation in the skin associated with transcutaneous allergen sensitization is one of the risk factors of this type of immunoglobulin isotype formation with high levels and high antigen‐affinity of IgE in early infancy. This speculation is also supported by the results shown in Figure 7B that the levels of OVA‐specific IgE were significantly higher at 6 months of age in infants with eczema history than those without such history (*P* < 0.0001). In this regard, it has been reported that IL‐4 plays a key role in class‐switching processes of *γ*1 → ε and high interleukin 4 (IL‐4) levels in the inflamed skin lesions can stimulate IgE production.^38^, ^39^ We reported previously that high‐affinity IgE, but not low‐affinity IgE, elicits degranulation signaling in mast cell line RS‐ATL8 cells.^28^ In experiments of mast cell degranulation by using various IgE clones of different affinities, Hjort et al^40^ also reported recently that human mast cell activation occurs with increased allergen‐affinity of IgE. Furthermore, a strong association between food sensitization and atopic dermatitis and/or skin barrier impairment has been reported.^9^, ^16^, ^17^, ^41^, ^42^, ^43^ We reported previously the presence of strong association between persistent eczema and high incidence of ovomucoid‐specific high‐affinity IgE in 14‐month‐old infants.^30^ The present longitudinal study of immunoglobulin isotype formation found low levels of IgG1, undetectable levels of IgG2 and high levels and high‐affinity IgE in the red circles infants, suggests that this type of immunoglobulin isotype formation is closely related to eczema history and could potentially be a biomarker of progression of food allergy. In contrast, it has been reported that low‐affinity IgE can compete with high‐affinity IgE for binding to Fcε receptors, resulting in the prevention of allergic reaction.^36^ The present data also suggest that well‐developed immunoglobulin isotype formation through oral food allergen sensitization found in the casein‐specific immunoglobulin isotype formation and in the black circles infants in group B with higher levels of OVA‐specific IgG1 associated with detectable IgG2 and low‐affinity IgE production, probably plays an important role in the development of oral tolerance.

Our study has certain limitations. This observational study was designed to analyze feeding type‐related allergen‐specific immunoglobulin isotype formation during the first 6 months of life to clarify the immunological background of food allergy and oral tolerance. The results showed two distinct patterns of allergen‐specific immunoglobulin isotype formation with low‐ and high‐affinity IgE, which could serve as predictors of future food allergy and/or oral tolerance. While the present study outlined the characteristic responses of casein‐ and OVA‐specific IgG1, IgG2, and IgE formation, it did not characterize other late responsive immunoglobulins, such as IgG4 and IgA, which are thought to play at least some roles in oral tolerance. Further studies of a larger sample size, longer follow‐up period (until at least 1 year of age) and firm diagnosis of food allergy at 1 year of age are needed to confirm our speculations and conclusions. In addition, we found no significant impact on the levels of OVA‐specific immunoglobulins in the feeding types (Figure 5 and Table S2). The results suggest that further studies on allergen sensitization including aeroallergen sensitization other than oral and cutaneous sensitization are needed to clarify the immunological background of food allergy in early infancy.

In conclusion, our longitudinal study identified two different patterns of allergen‐specific immunoglobulin isotype formation with high‐ and low‐affinity IgE in the first 6 months of life of normal breast‐, formula‐ and mixed‐feeding infants with and without eczema.

## CONFLICT OF INTERESTS

The authors declare that there are no conflict of interests.

## AUTHOR CONTRIBUTIONS

MI and HK formulated the research hypothesis; MI, WS, MS, YO, KS, and KK conducted the experimental work, analyzed the data, and interpreted the results; KA and MI conducted the statistical analysis; MI, YO, and HK drafted the manuscript; and HS, YO, LY, and SK critically revised the manuscript.

## Supporting information

Supporting information
