# Supplementary material for: Trajectories of class–switching‐related egg and cow's milk allergen‐specific immunoglobulin isotype formation and its modification by eczema with low‐ and high‐affinity immunoglobulin E during early infancy
Source: Immun Inflamm Dis. 2019 Mar 11;7(2):74–85. doi: 10.1002/iid3.245 (PMC6485699; doi:10.1002/iid3.245)
Supplement: Supplementary file 1 — Supporting information [file IID3-7-74-s001.pdf]

Supplemental information

Trajectories of class-switching related egg and cow's milk allergen-specific immunoglobulin isotype formation and and its modification by eczema with low- and high-affinity IgE during early infancy

Makoto Irahara[1,2], Wakako Shinahara[1], Mayumi Sugimoto[2], Yukiko Ogawa[3], Keiji Shitsukawa[4], Kenji Kubota[1], Limin Yang[5], Yukihiro Ohya[5], Hirohisa Saito[5], Shoji Kagami[2], Kokichi Arisawa[6] & Hiroshi Kido*[1]

[1]Division of Enzyme Chemistry, Institute for Enzyme Research; [2]Department of Pediatrics; [3]Department of Pediatrics Tokushima Prefecture Naruto Hospital, Tokushima, Japan; [4]Department of Obstetrics and Gynecology, Tokushima Prefecture Naruto Hospital, Tokushima, Japan; [5]National Center for Child Health and Development, Tokyo, Japan; [6]Department of Preventive Medicine, Graduate School of Medicine, Tokushima University, Tokushima, Japan.

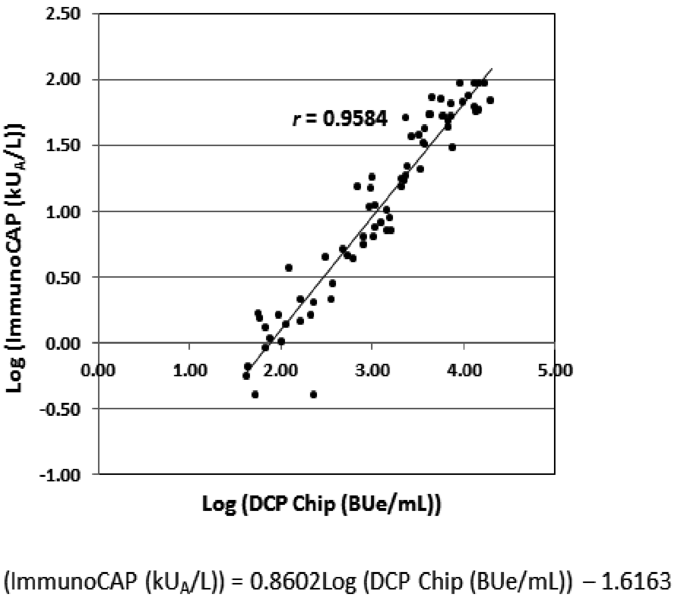

Figure S1 Correlation coefficient and equation of casein-specific IgE levels between UniCAP (kU_A/mL) and DCP microarray (BUe/mL).

Table S1. Serial changes in feeding type-related production of casein-specific immunoglobulins in breast, formula and mixed feeding infants from birth to 6-months of age. Data are median (IQR).

**Casein- specific immunoglobulins**	**CB**	**2 months of age**			**4 months of age**			**6 months of age**		
		**Breast (n=31)**	**Mixed (n=47)**	**Formula (n=6)**	**Breast (n=31)**	**Mixed (n=47)**	**Formula (n=6)**	**Breast (n=31)**	**Mixed (n=47)**	**Formula (n=6)**
**IgE (BUe/mL)**	0.01 (0.01-41)	0.01 (0.01-0.01)	0.01 (0.01-30)	21 (3-142)	0.01 (0.01-0.01)	0.01 (0.01-88)	84 (33-144)	0.01 (0.01-0.01)	37 #,† (0.01-159)	149 (46-170)
			P=0.004[a]			*P*=0.002[a]			*P*=0.001[a]	
**IgG1 (BUg1/mL)**	1363(527-2787)	729 # (335-956)	2134 (438-3959)	3740 (3554-4840)	282 #† (173-689)	2380 (795-4496)	3929 (3529-4800)	212 #† (151-391)	2308 (699-4812)	4560 (3390-6690)
			P=0.001[a]			*P*<0.001[a]			*P*<0.001[a]	
**IgG2 (BUg2/mL)**	78 (61-166)	58 # (50-72)	94 (65-286)	379 (180-432)	56 # (50-67)	114 (65-266)	484 (179-710)	53 # (50-63)	108 (70-408)	449 (182-748)
			P<0.001[a]			*P*<0.001[a]			*P*<0.001[a]	
**IgG3 (BUg3/mL)**	50 (50-50)	50 (50-50)	56 (50-128)	131 (109-292)	50 (50-50)	65 (50-139)	185 (84-396)	50 (50-50)	62 (50-139)	128 (70-372)
			P<0.001[a]			*P*<0.001[a]			*P*<0.001[a]	
**IgG4 (BUg4/mL)**	88 (51-226)	56 (50-82)	67 (50-82)	80 (50-90)	54 (50- 81)	52 (50-86)	81 (70-288)	50 (50-62)	58 (50-88)	150 (51-566)
			P=0.42[a]			*P*=0.04[a]			*P*=0.008[a]	
**IgA (Bua/mL)**	3 (3-5)	3 (3-6)	19 (4-49)	40 (30-76)	3 (3-5)	12 (4-49)	33 (18-70)	3 (3-10)	11 (5-40)	26 (12-92)
			P<0.001[a]			*P*<0.001[a]			*P*=0.001[a]	

Differences among three feeding type groups were tested by the Kruskal-Wallis test and [a]*P*-value less than 0.05 was considered significant. Significant difference between the two different time points shown in Figure 2: #, vs CB, and †, vs 2-months of age (Wilcoxon signed-rank test with Bonferroni correction. *P*-value <0.01was judged as significant).

Table S2. Serial changes in production of feeding type-related OVA-specific immunoglobulins in infants on breast, formula and mixed feeding from birth to 6-months of age.

OVA- specific immunoglobulins	CB	2 months of age			4 months of age			6 months of age		
		Breast (n=31)	Mixed (n=47)	Formula (n=6)	Breast (n=31)	Mixed (n=47)	Formula (n=6)	Breast (n=31)	Mixed (n=47)	Formula (n=6)
**IgE level (BUe/mL)**	0.01 (0.01-80)	0.01 # (0.01-41)	0.01 # (0.01-0.01)	0.01 (0.01-3)	0.01 (0.01-91)	0.01 (0.01-10)	0.01 (0.01-0.01)	7 † (0.01-447)	0.01 †§ (0.01-170)	0.01 (0.01-0.01)
			P=0.33[a]			*P*=0.07[a]			*P*=0.08[a]	
**IgG1 level (BUg1/mL)**	527 (240-1136)	266 # (165-363)	152 # (125-288)	305 (122-344)	160 (121-523)	180 (111-400)	141 (105-175)	176 § (119-1008)	408 †§ (156-1609)	153 (107-319)
			P=0.05[a]			*P*=0.39[a]			*P*=0.17[a]	
**IgG2 level (BUg2/mL)**	137 (71-276)	82 # (61-96)	68 # (60-93)	80 (56-162)	61 #† (56-70)	61 # (56-76)	80 (65-116)	59 (54-74)	63 (59-72)	61 (58-64)
			P=0.61[a]			*P*=0.26[a]			*P*=0.16[a]	
**IgG3 level (BUg3/mL)**	50 (50-50)	50 (50-50)	50 (50-50)	50 (50-50)	50 (50-50)	50 (50-50)	50 (50-50)	50 (50-50)	50 (50-50)	50 (50-50)
			P=1.0[a]			*P*=0.27[a]			*P*=0.33[a]	
**IgG4 level (BUg4/mL)**	540 (113-1364)	113 (56-341)	70 (50-220)	115 (50-472)	56 (50-88)	52 (50-71)	58 (51-102)	50 (50-52)	50 (50-53)	50 (50-75)
			P=0.30[a]			*P*=0.32[a]			*P*=047[a]	
**IgA level (Bua/mL)**	3 (3-4)	5 (3-7)	3 (31-5)	4 (4-4)	4 (3-8)	3 (3-5)	3 (3-5)	5 (3-10)	5 (3-13)	3 (3-5)
			P=0.05[a]			*P*=0.17[a]			*P*=0.43[a]	

Data are median (interquartile range). [a]*P*-value for the difference among the three feeding type groups analyzed by the Kruskal-Wallis test. #, vs CB, †, vs 2-months of age, § vs 4-months of age (Figure 4), by Wilcoxon signed-rank test with Bonferroni correction. *P*-value <0.01 was considered significant.

Table S3. OVA-specific IgE, IgG1 levels and IC_{50} values of IgE against OVA in each red and black circles infants shown in Figure 6F.

Subject number	Feeding type	OVA-specific immunoglobulins			Circles
		IgE IC_{50} (nM)	IgE level (BUe/mL)	IgG1 level (BUg1/mL)	
2	Mixed	25.4	435	4553	black
5	Mixed	57.2	244	3081	black
10	Mixed	9.2	771	1288	red
13	Mixed	75.3	720	5976	black
23	Breast	5.8	205	782	red
34	Mixed	6.5	1259	1002	red
36	Mixed	84.9	276	279	red
45	Mixed	61.3	222	2416	black
47	Mixed	1.9	825	791	red
49	Breast	8.7	447	3189	black
64	Breast	78.0	452	6143	black
65	Breast	9.1	265	1272	red
71	Breast	8.3	1176	738	red
73	Mixed	47.3	470	2145	black
74	Mixed	15.4	917	594	red
76	Mixed	4.6	929	522	red
80	Breast	6.7	1567	410	red
81	Breast	5.3	504	480	red
82	Breast	6.3	904	435	red
83	Breast	18.8	1517	168	red
